# Supplementary material for: Combination Therapy versus Monotherapy in the Treatment of Stenotrophomonas maltophilia Infections: A Systematic Review and Meta-Analysis
Source: Antibiotics (Basel). 2022 Dec 9;11(12):1788. doi: 10.3390/antibiotics11121788 (PMC9774194; doi:10.3390/antibiotics11121788)
Supplement: Supplementary file 1 [file antibiotics-11-01788-s001.zip › antibiotics-2009831-supplementary.pdf]

Supplementary materials

Table

Table S1. Search algorithms.

Table S2. Quality assessments of case reports and case series

Table S3. Description of characteristics of included studies of combination treatment of *S. maltophilia* infection.

Table S4. Description of characteristics of cohort studies included in the systematic review and meta-analysis**.**

Figure

Figure S1. Summarized risk of bias of included studies using ROBINS-I tool

Figure S2. The funnel plot of included studies in the meta-analysis

Figure S3. The graph from Egger's test of included studies in the meta-analysis

TableS1. Search algorithms.

Database	No	Step search algorithm	Items found
PubMed Central	#1	Search *Stenotrophomonas*	13986
	#2	Search Mortality	1,708,126
	#3	Search Therapeutics	1,115,408
	#4	Search Anti-bacterial agents	283,485
	#5	Search Therapeutics OR Anti-bacterial agents	1,318,731
	**#6**	**Search *Stenotrophomonas* AND Mortality AND (Therapeutics OR Anti-bacterial agents)**	**2,992**
Cochrane Library	#1	Search *Stenotrophomonas*	71
	#2	Search Mortality	106,143
	#3	Search Therapeutics	387,835
	#4	Search Anti-bacterial agents	11,917
	#5	Search Therapeutics OR Anti-bacterial agents	338,259
	**#6**	**Search *Stenotrophomonas* AND Mortality AND (Therapeutics OR Anti-bacterial agents)**	**17**
EMBASE	#1	Search *Stenotrophomonas*	9,317
	#2	Search Mortality	1,787,666
	#3	Search Therapeutics	477,741
	#4	Search Anti-bacterial agents	22,095
	#5	Search Therapeutics OR Anti-bacterial agents	344,732
	**#6**	**Search *Stenotrophomonas* AND Mortality AND (Therapeutics OR Anti-bacterial agents)**	**1,741**
SCOPUS	#1	Search *Stenotrophomonas*	7,164
	#2	Search Mortality	1,663,755
	#3	Search Therapeutics	140,085
	#4	Search Anti-bacterial agents	278,743
	#5	Search Therapeutics OR Anti-bacterial agents	324,735
	**#6**	**Search *Stenotrophomonas* AND Mortality AND (Therapeutics OR Anti-bacterial agents)**	**278**
Clinicaltrial.gov	#1	Search *Stenotrophomonas*	3
	#2	Search Mortality	1,925
	#3	Search Therapeutics	102207
	#4	Search Anti-bacterial agents	2,751
	#5	Search Therapeutics OR Anti-bacterial agents	103,907
	**#6**	**Search *Stenotrophomonas* AND Mortality AND (Therapeutics OR Anti-bacterial agents)**	**2**
Open Grey	#1	Search *Stenotrophomonas*	4
	#2	Search Mortality	1,395
	#3	Search Therapeutics	38
	#4	Search Anti-bacterial agents	0
	#5	Search Therapeutics OR Anti-bacterial agents	471
	**#6**	**Search *Stenotrophomonas* AND Mortality AND (Therapeutics OR Anti-bacterial agents)**	**0**

Table S2. Quality assessments of case reports and case series

Author, year	Item 1[a]	Item 2[b]	Item 3[c]	Item 4[d]	Item 5[e]	Item 6[f]	Item 7[g]	Item 8[h]
Munter (1998) [15]	Y	Y	Y	Y	N	N	Y	Y
Kim (2001) [16]	Y	Y	Y	N	N	N	Y	Y
Wood (2010) [17]	Y	Y	Y	N	N	N	Y	Y
Holifield (2011) [18]	Y	Y	Y	N	N	N	Y	Y
Mori (2014) [19]	Y	Y	Y	Y	N	N	Y	Y
Reynaud (2015) [20]	Y	Y	Y	Y	N	N	Y	Y
Mojica (2016) [21]	Y	Y	Y	N	N	N	Y	Y
Subhani (2016) [12]	Y	Y	Y	N	N	N	Y	Y
Kaito (2018) [22]	Y	Y	Y	Y	N	N	Y	Y
Payen (2019) [11]	Y	Y	Y	Y	N	N	Y	Y
Andrei (2020) [23]	Y	Y	Y	N	N	N	Y	Y
Khanum (2020) [10]	Y	Y	Y	N	N	N	Y	Y

[a]Does the patient(s) represent the whole experience of the investigator (center)?

[b]Was the exposure adequately ascertained?

[c]Was the outcome adequately ascertained?

[d]Were other alternative causes that may explain the observation ruled out?

[e]Was there a challenge/rechallene phenomenon?

[f]Was there a dose-response effect?

[g]Was follow-up long enough for outcome to occur?

[h]Is the case(s) described with sufficient details to allow other investigators to replicate the research or to allow practitioner make inference related to their own practice?

	1. Bias due to judgment confounding	2. Bias in selection of participants	3. Bias in measurement of interventions	4. Bias due to deviations from intended interventions	5. Bias due to missing data	6. Bias in measurement of outcomes	7. Bias in selection of reported results	Overall
Shah et al (2019)	Low	Moderate	Low	Moderate	Low	Low	Low	Moderate
Guerci et al (2019)	Moderate	Moderate	Low	Low	Moderate	Low	Moderate	Serious
Araoka et al (2017)	Moderate	Low	Moderate	Low	Low	Low	Low	Moderate
Muder et al (1996)	Serious	Low	Moderate	Low	Low	Low	Moderate	Serious

Figure S1 Summarized risk of bias of included studies using ROBINS-I tool

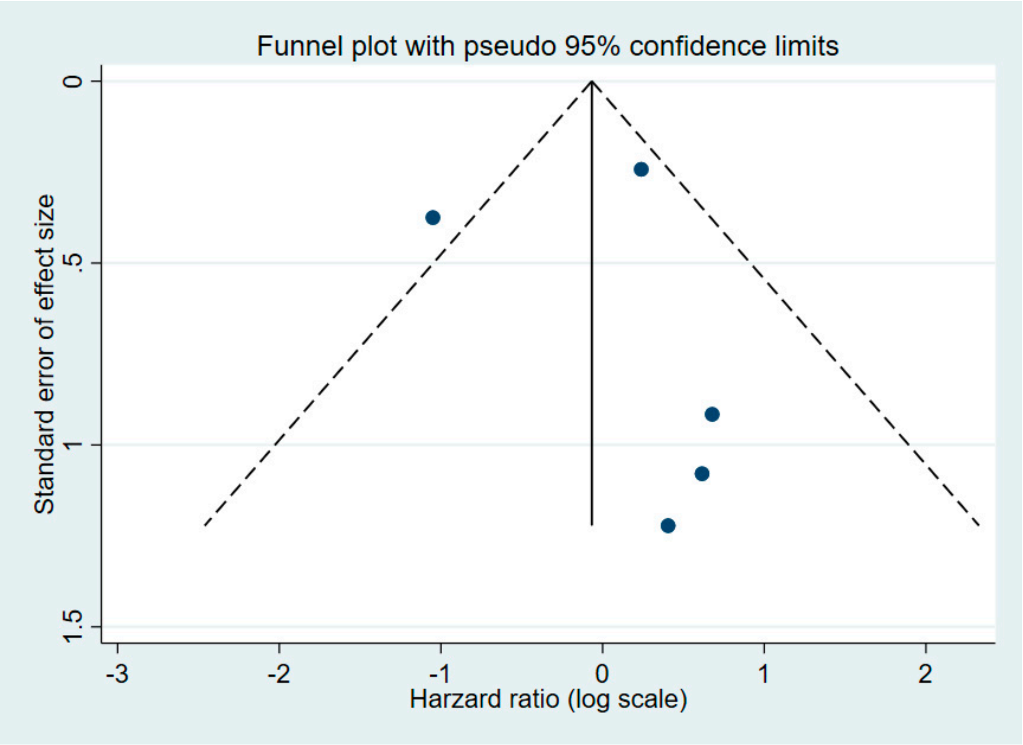

Figure S2. The funnel plot of included studies in the meta-analysis

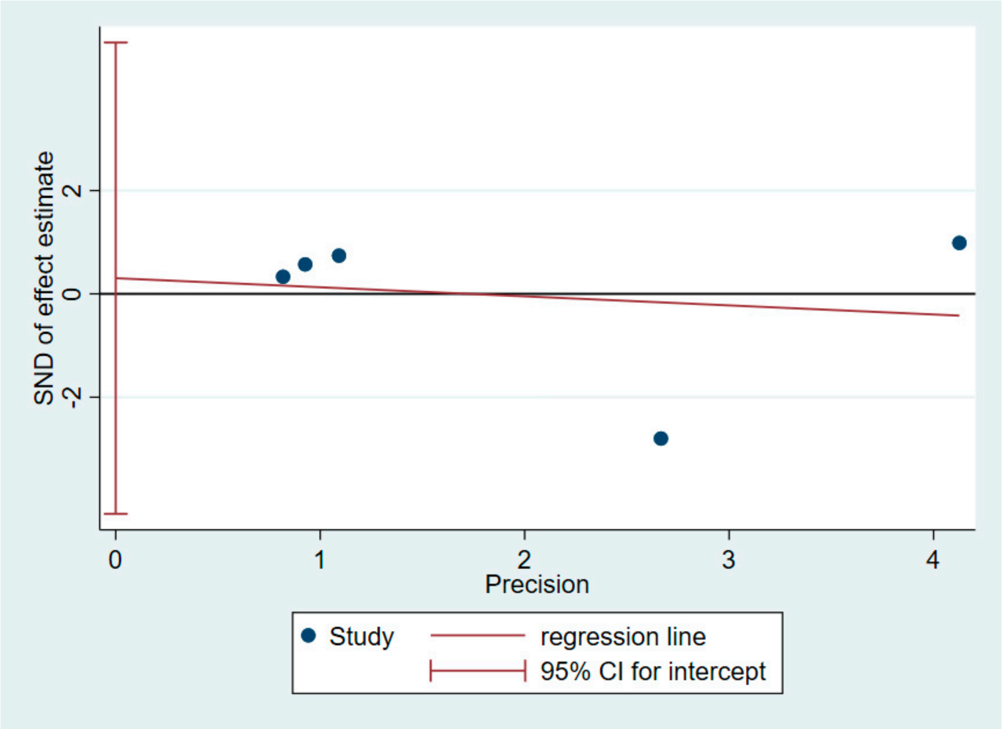

Figure S3. The graph from Egger's test of included studies in the meta-analysis

Table S3. Description of characteristics of included studies of combination treatment of *S. maltophilia* infection.

Author/ Year	Region	Design	Sample size	Sex	Age (years)	Infection	Antimicrobial susceptibility	Details of antimicrobials		Treatment duration (days)	Follow up time (days) (n)	Outcome
								Combined antimicrobials	Dosage regimens			
Robert, 1996	USA	Cohort	18	N/A	23-85	Bacteremia	TMP-SMX: S (91%)	2 or more of these agents<br>-TMP-SMX<br>-third-generation cephalosporin<br>-extended-spectrum penicillin	N/A	N/A	N/A	died 18%
Munter, 1998	Isreael	Case report	1	F	69	Infective endocarditis	TMP-SMX: S<br>Ciprofloxacin: S	TMP-SMX+ CIP	N/A	40	N/A	Died
Jae-Han, 2001	Korea	Case report	1	M	44	Infective endocarditis	TMP-SMX: S<br>Tobramycin: S<br>Gentamicin: S<br>Ofloxacin: S<br>Ciprofloxacin: I	TMP-SMX+ Tobramycin	-TMP-SMX: 400 mg/80 mg IV q 8 hr<br>-Tobramycin: 1.7 mg/kg IV q 8 hr	42	60	Clinical response
Wood, 2010	USA	Case report	1	M	28	VAP	TMX-SMX: S<br>Doxycycline: S<br>Minocycline: S<br>Colistin: S	TMX-SMXthen change todoxycycline+ Aerosolized COL	-TMP-SMX : TMP 4 mg/kg IV q 6 hr<br>-Doxycycline: 100 mg IV q 12 hr<br>-Aerosolized COL: 150 mg NB q 12 hr	14	33	- Clinical response<br>- Microbiological response
Karintha, 2011	USA	Case report	1	M	41	Keratitis	N/A	Gatifloxacin ED + polymyxin/trimethoprim ED	-Gatifloxacin0.3% ED: q 2 hr to left eye<br>-polymyxin/trimethoprim 0.1% ED: q 2 hr to left eye	10	N/A	- Clinical response<br>- Microbiological response
Mori, 2014	Japan	Case series	8	F (3), M (5)	42-62	Hemorrhagic pneumonia	TMP-SMX: S (80-90%)<br>fluoroquinolone: S (50-70%)	TMP-SMX+ fluoroquinolone	-TMP-SMX: high dose<br>-Fluoroquinolone: N/A	1-16	N/A	died 100%
Reynaud, 2015	France	Case report	1	F	81	Infective endocarditis	N/A	TMP-SMX+ Moxifloxacin	N/A	2	N/A	Died
Maria, 2016	USA	Case report	1	M	19	Bacteremia	Minocycline: I	CAZ/avibactam+ Aztreonam	-CAZ/avibactam: 2.5 g IV q 8 hr<br>-Aztreonam: 2 g IV q 8 hr	48	N/A	- Microbiological response
Subhani, 2016	India	Case series	28	M (20)<br>F (8)	22-78	Infective endocarditis	N/A	CHL+KAN+COL<br>CAR+GEN+KAN,<br>CHL+PEN+POL<br>CAR+GEN+ TMP-SMX<br>STR+PEN<br>CAR+AMK+ TMP-SMX<br>CAR+KAN+ TMP-SMX<br>POL+ TMP-SMX<br>GEN+CHL<br>TIC+MOX+ TMP-SMX<br>TMP-SMX +AMC+GEN<br>CAZ+GEN+ TMP-SMX<br>CIP+GEN	N/A	42 (1), N/A (27)	N/A (27), 60 (1)	- 67.86% cured<br>- 28.57% died<br>- 3.57% N/A

Author/ Year	Region	Design	Sample size	Sex	Age (years)	Infection	Antimicrobial susceptibility	Details of antimicrobials		Treatment duration (days)	Follow up time (days) (n)	Outcome
								Combined antimicrobials	Dosage regimens			
								TIM+ TMP-SMX<br>TZP+GEN<br>CAZ+GEN+CIP+ TMP-SMX CAZ+AMK+CIP then TIM+ TMP-SMX +COL<br>CIP+CHL<br>TIM+ TMP-SMX<br>VAN+GEN<br>VAN+GEN<br>LVX+ TMP-SMX<br>FEP+CIP+ TMP-SMX<br>TMP-SMX +GEN<br>TMP-SMX +TIM<br>TMP-SMX +CAZ<br>TMP-SMX +CIP+TZP				
Araoka, 2017	Japan	Cohort	14	M (85.71 %)	23-72	Bacteremia	TMP-SMX: S (94%)	N/A	N/A	N/A	30	died 50%
Satoshi, 2018	Japan	Case report	1	M	28	Bacteremia, Pneumonia	N/A	TMP-SMX+CIP	N/A	18	N/A	Died
Payen, 2019	France	Case series	4	N/A	56-83	VAP (2), Peritonitis (1)	N/A	CIP+ CAZ,tigecycline+colimycin	N/A	14	176	- 100% had clinical response<br>- 100% had microbiological response
Shah, 2019	USA	Cohort	38	M (29%)	18-89	Pneumonia	TMP-SMX: S (8%)<br>Fluoroquinolone:S (13%)<br>Minocycline: S (0%)<br>Ceftazidime: S (1%)	TMP-SMX + LVX,<br>TMP-SMX + CIP,<br>TMP-SMX + moxifloxacin,<br>TMP-SMX + minocycline,<br>TMP-SMX + CAZ,<br>LVX + minocycline,<br>LVX + CAZ,<br>CIP + minocycline,<br>CIP + CAZ,<br>minocycline + CAZ	N/A	N/A	N/A	died 39.47%
Guerci, 2019	France	Cohort	167	M (69.9 %)	56-74	Pneumonia	TMP-SMX: S (88.1%)<br>TIM (73.3%)	N/A	N/A	7	N/A	died 37.72%
Stefan, 2020	Romania	Case report	1	F	61	Severe pneumonia with pulmonary hemorrhage	TMP-SMX: S<br>Levofloxacin: I	COL + TMP-SMX	N/A	7	300	- Clinical response<br>- Microbiological response
Khanum, 2020	Pakistan	Case series	2	F (1), M (1)	60, 35	Meningitis	TMP-SMX: S<br>Colistin: S	COL (polymyxin E) + TMP-SMX,<br>TMP-SMX + CAZ	-polymyxin E: 3 million units q 8 hr along with intrathecal polymyxin E: 300,000 IU/day,<br>-TMP-SMX: TMP15m/kg/d in 3 divided doses,<br>-CAZ: 2 gm IV q 8 hr	21	N/A	- 100% had clinical improvement<br>- 100% had CSF culture negative

Abbreviations: ED; eye drop, F; female, I: intermediate, M; male, N/A; not applicable, NB; nebulize, R; resistant, S; susceptible; AMC, ampicillin; AMK, amikacin; CAR, carbenicillin; CAZ, ceftazidime; CHL, chloramphenicol; CIP, ciprofloxacin; COL, colistin; FEP, cefepime; GEN, gentamicin; KAN, kanamycin; LVX, levofloxacin; MOX, moxalactam; PEN, penicillin; POL, polymyxin; STR, streptomycin; TIC, ticarcillin; TIM, ticarcillin/clavulanic acid; TMP-SMX; trimethoprim-sulfamethoxazole; TZP, piperacillin/tazobactam; VAN, vancomycin

Table S4. Description of characteristics of cohort studies included in the systematic review and meta-analysis**.**

**characteristic**	**Included cohort studies**			
	**Shah et al (2019)**	**Guerci et al (2019)**	**Araoka et al (2017)**	**Muder et al (1996)**
region	USA	France	Japan	USA
Study design	Cohort study	Cohort study	Cohort study	Cohort study
Duration of study (year)	6	5	2	3
Sample size (n)	252	282	20	91
Characteristic of participants	Patient age: 18-89 years old with *S.maltophilia*pneumonia	Patient age ≥18 years old who with *S.maltophilia*pneumonia	Patient age:23-85 years old with *S.maltophilia*bacteremia infection	Patient age:23-85 years old with *S.maltophilia*bacteremia infection
Outcome	Primary outcome**:**	Primary outcome**:**	Primary outcome**:**	Primary outcome**:**
	7-day clinical response	Time to in-hospital death	*In vitro* effects of TMP-SMXcombined with other antimicrobial agents against *S. maltophilia*strains and clinical efficacy and 30-day mortality	clinical outcome and survival rate.
	Secondary outcomes:	Secondary outcomes:	Secondary outcomes	Secondary outcomes
	Microbiological cure	Microbiologic effectiveness	-N/A	-N/A
	30-day recurrence of infection	Antimicrobial therapeutic modilities		
	Hospital length of stay			
	Infection-related length of stay			
	ICU length of stay			
	30-day infection-related mortality			

characteristic	Included cohort studies			
	Shah et al (2019)	Guerci et al (2019)	Araoka et al (2017)	Muder et al (1996)
	30-day all-cause mortality<br>Resistance development			
Treatment details	Monotherapy<br>TMP-SMX<br>Levofloxacin<br>Ciprofloxacin<br>Moxifloxacin<br>Minocycline<br>Ceftazidime<br>Combination therapy<br>TMP-SMX + Levofloxacin<br>TMP-SMX + Ciprofloxacin<br>TMP-SMX +Moxifloxacin<br>TMP-SMX +Minocycline<br>TMP-SMX + Ceftazidime<br>Levofloxacin + Minocycline<br>Levofloxacin + Ceftazidime<br>Ciprofloxacin + Minocycline<br>Ciprofloxacin + Ceftazidime<br>Minocycline + Ceftazidime	Monotherapy<br>TMP-SMX<br>Levofloxacin<br>Ciprofloxacin<br>Ticarcillin/clavulanate<br>Ceftazidime<br>Minocycline<br>Colistin<br>Rifampicin<br>Tigecycline<br>Combination therapy<br>N/A	Monotherapy<br>TMP-SMX<br>Combination therapy<br>TMP-SMX + fluoroquinolone	Monotherapy<br>TMP-SMX<br>Third-generation cephalosporin<br>Extended-spectrum penicillin<br>Combination therapy<br>Receiving more than 1 of monotherapy agents
Comorbidity of participants	Cancer receiving chemotherapy<br>HSCT	COPD<br>Chronic respiratory insufficiency	Hematological malignancies<br>Solid tumors	Malignancy<br>Cardiac disease

characteristic	Included cohort studies			
	Shah et al (2019)	Guerci et al (2019)	Araoka et al (2017)	Muder et al (1996)
	Solid organ transplant<br>HIV infection	Cystic fibrosis<br>Hypertension<br>Congestive heart failure<br>Receiving dialysis<br>Liver cirrhosis<br>Insulin requiring diabetes<br>Severe neurologic disability		Chronic pulmonary disease<br>Receipt of a transplant<br>Chronic liver disease<br>Receiving dialysis<br>HIV infection
Effect size (95% CI)	(A) = 1.85 (0.75-4.98)<br>(B) = 1.97 (0.96-4.55)	1.27 (0.88-1.83)	1.5 (0.43-5.22)	0.35 (0.08-3.18)
Immunocompromised population (%)	19.8	37.4	100	97.8
Polymicrobial (%)	54.4	58.4	0	40
Age of exposure group (year)	62a	65 (±9)[b]	60.5[a]	N/A
Male (%)	62.3	69.9	85.71	N/A
Severity score	APACHE II score	SOFA score	Pitt score	Severity score

a = mean age (year), b = mean (SD), N/A; not applicable; (A)= 30-day infection related mortality; (B)= 30-day all cause mortality, COPD; Chronic obstructive Pulmonary disease, HIV; Human immunodeficiency virus, HSCT; hematopoietic stem cell transplantation, TMP-SMX; sulfamethoxazole-trimethoprim.
